# Agroecological coffee management increases arbuscular mycorrhizal fungi diversity

**DOI:** 10.1371/journal.pone.0209093

**Published:** 2019-01-08

**Authors:** Paulo Prates Júnior, Bruno Coutinho Moreira, Marliane de Cássia Soares da Silva, Tomas Gomes Reis Veloso, Sidney Luiz Stürmer, Raphael Bragança Alves Fernandes, Eduardo de Sá Mendonça, Maria Catarina Megumi Kasuya

**Affiliations:** 1 Departamento de Microbiologia, Universidade Federal de Viçosa, Viçosa, Minas Gerais, Brazil; 2 Colegiado de Engenharia Agronômica, Universidade Federal do Vale do São Francisco, Petrolina, Pernambuco, Brazil; 3 Departamento de Ciências Naturais, Universidade Regional de Blumenau, Blumenau, Santa Catarina, Brazil; 4 Departamento de Solos, Universidade Federal de Viçosa, Viçosa, Mina Gerais, Brazil; 5 Departamento de Produção Vegetal, Universidade Federal do Espírito Santo, Alegre, Espírito Santo, Brazil; Universita degli Studi del Piemonte Orientale Amedeo Avogadro, ITALY

## Abstract

Agroecology aims to maintain ecosystem services by minimizing the impact of agriculture and promoting the use of biological potential. Arbuscular mycorrhizal fungi (AMF) are elements which are key to improving crop productivity and soil quality. It is pertinent to understand how agricultural management in the tropics affects the AMF spatio-temporal community composition, especially in crops of global importance, such as coffee (*Coffea arabica* L.). Soil and root samples were collected from three localities under three management systems (agroecological, conventional and forest fragment), during the phenological stages of coffee (flowering, grain filling, harvesting). Spores were extracted for morphological identification and molecular community analysis by PCR–DGGE. Dendrograms were prepared and the bands were sequenced and analyzed by bioinformatics. No differences were observed in the richness of morphospecies between management systems, localities and period, but little is known about tropical species. Molecular analysis showed that the agroecological management system was similar to natural forest and with a higher diversity indices than conventional management. Locality and period of sample affect AMF community composition. It is necessary to associate classical taxonomic evaluations with molecular biological techniques because different approaches can lead to different outcomes. This study contributes to the understanding of the impact of agriculture management systems on AMF and provides evidence that agroecology is a management system applicable to sustainable coffee production.

## Introduction

The practice of conventional agriculture has increased food production, but produced negative effects in ecosystem services, affecting nutrient cycling, climate regulation, pest and disease control, soil stabilization and carbon sequestration [1–3]. As a result, agroecology-based agriculture has received attention on account of the adoption of low-input practices and development of more sustainable agro-food systems, based on the maintenance of biodiversity and ecosystem services [4–6]. Agroecology aims to provide a framework focused on food production to attend increasing global demand and on the conservation of natural resources, promoting food sovereignty and food and nutrition security [7,8]. Some of the current environmental concerns in agriculture are related to initiatives aimed at reducing excessive use of synthetic fertilizers and pesticides [9,10], improving nutrient management, preventing soil erosion, and maximizing the functions of soil microorganisms [11,12].

Arbuscular mycorrhizal fungi (AMF) are soil fungi that establish a symbiotic relationships with over 70% of plant species [13,14], and they represent elements which are key to agricultural productivity and biogeochemical process [15]. These fungi play an important ecological role in management practices in low-input agricultural systems [16,17]. AMF have multifunctional roles in natural and agricultural systems where they are involved in several ecosystem services [12,15,18]. Moreover, they are influenced by factors related to agricultural management [11,17], such as fertilization and the availability of nutrients (e.g. P and Zn), liming, soil aggregation [10], plant intercropping and the use of agrochemicals, among others, that impact AMF community composition parameters [19]. Studies have demonstrated that AMF abundance and diversity decreases under high-input conventional agriculture systems [20–22]. Therefore, it is relevant to understand how the agroecology-based agricultural management practices in tropical soils influence AMF community composition, especially when associated with crops of global importance, such as coffee.

Coffee (*Coffea arabica* L. and *C*. *canephora* Pierre—Rubiaceae) is grown in Africa, Asia and Latin America, and is a valuable commodity surpassed only by oil [19]. Coffee plantations represent a significant source of income in many parts of the world and competent management is critical to a rapidly growing population in the tropics [23,24] and increasing world demand. Coffee crops differ from region to region in the world [25] ranging from low [26] to high input practices [27]. Brazil stands out as the largest producer and is the second largest consumer of coffee, and the state of Minas Gerais is responsible for 67.8% of the area under cultivation in the country [28].

Coffee seedlings are highly dependent on mycorrhizal association [29], especially in highly weathered soils with low natural fertility as tropical soils [23,30]. Under field conditions, adult coffee plants are associated with a diverse community of AMF [21,22,31,32] and a total of 70 AMF species have been reported to be associated with coffee plants [33]. A review of studies published on the interaction between AMF and coffee plants shows that most studies have focused on the use of AMF as biofertilizers in the improvement of crop growth and the establishment and assessment of AMF taxonomic diversity associated with coffee plants [33]. However, no studies have as yet focused on the composition and dynamics of AMF community in coffee plantations over space and time, which is the first step towards management of AMF communities aimed at improving the quantity and quality of coffee production [12,34].

Spore abundance and mycorrhizal colonization can be higher in intercropping coffee compared to non-shaded coffee [31,35]and the presence of weeds and fertilization appears to influence AMF community composition [21,36]. Studies have suggested that adoption of more sustainable management systems in agriculture such as agroforestry and low input practices support a higher abundance and diversity of AMF community when compared to conventional systems [9,26,36]. Therefore, to understand the potential beneficial effects of the AMF community over space and time on soil quality evaluations based on microorganism aspects related to diversity and composition must be carried out. To accomplish this classical taxonomic evaluations associated with modern molecular tools are required [36–38].

Considering the importance of agroecological systems to agricultural sustainability in tropical systems, the multi-functional role of AMF and the importance of coffee as a crop in several parts of the world, the aim of this study was to test if coffee agroecological systems increase the diversity and abundance of AMF, in space and time, in comparison with conventional agricultural practices. We tested the hypothesis that the AMF community associated with coffee plants under an agroecological management system is more diverse than conventional management systems.

## Materials and methods

### Study sites

The study sites were located in Araponga, in the state of Minas Gerais, Brazil. This area has a tropical climate with an average annual temperature of 18°C, and average annual rainfall of 1,500 mm, with a dry season from May to August, and terrain topography with steep slopes of 20–45% and average altitude of 1,040 m [31]. Soils are mainly Red-Yellow Oxisols, deep, weathered and well-drained, acid and with low natural fertility [39]. Small farms predominate in the area, and common management practices include turning the soil slightly using hand tools and growing coffee (*Coffea arabica* L.) often cultivated intercropped with corn and beans. Other agricultural systems include pasture, cassava and sugarcane plantations [40].

Three localities, denominated herein as Z1, Z2, and Z3, were selected within the study area. From each locality, three sites were selected for sampling soils and roots: forest, coffee plantations under both conventional and agroecological management systems (Table 1). Forest fragments (F) included areas of tropical rain forest under natural secondary succession (13 to 30 years) pertaining to the Atlantic Rain Forest floristic Domain. Coffee plantations under agroecological management (A) were systems that adopted green manure fertilization, reduction of fertilizers application, and consortium with other crops. Conventional coffee plantations (C) included areas grown with no consortium crops that have received high doses of fertilizers for the last 30 years.

**Table 1:** Characteristics of forest fragment (F), agroecological (A) and conventional (C) coffee management and in three localities (Z1, Z2 and Z3).

Characteristics of areas	Management and Locality								
	F Z1^a^	A Z1^a^	C Z1^a^	F Z2^a^	A Z2^b^	C Z2^a^	F Z3^a^	A Z3^a^	C Z3^a^
**Management age**	In natural recovery approx. 13 yrs	Approx. 11 yrs	Approx. 40 yrs	Aprox. 30 yrs	Largest share with 14 and with the least seven yrs	Approx. 8 yrs	Approx. 30 yrs	Aprox. 20 yrs	Approx. 30 yrs
**Total area (ha)**	Approx. 0.6 ha	Approx. 0.1 ha	Approx. 0.56 ha	>3 ha	0.62 ha	Approx. 1.3 ha	>2 ha	Approx. 1.3 ha	Approx. 4 ha
**Fertilization**	N/A	150 g / plant or NPK 20.5.20 3X per yr 20-0-20	150 g / plant or NPK 20.5.20 3X per yr 20-0-20	N/A	80 g / NPK plant 05/02/20 2X yrs + 2.5 kg of poultry litter + foliar fertilization with micronutrients	150 g / NPK 20-05-20 plant or 3x yrs + foliar fertilization (zinc sulfate, potassium chloride and boric acid).	N/A	50g / NPK 05/20/20 plant, manure and lime once every three yrs (100 g/ den).	150 g / plant or NPK 20.5.20 3X yrs 20-0-20
**Consortium**	N/A	Green manure: lablab (*Dolichos lablab L*.*)* for 3 years, followed by 2 years of planting beans (*Phaseolus vulgaris* L).	No	N/A	Banana, jack bean (*Canavalia ensiformis* (L) CD) and 12 tree species, e.g: *Solanum macropus* Dunal, *Annona crassiflora* Mart., *Cecropia pachystachya* Trécul	No	N/A	Main species: banana (*Musa* sp.*)*, *Feuilleea uruguensis* Kuntze *Rollinia silvatica* Mart., *Jacaranda macrantha* Cham., *Eucalyptus* sp.	None but there was, intercropped with maize and beans between the lines.

Source

^a^Information gathered by the farmer's family

^b^Alves et al [41] and information gathered by the farmer's family.

The criterion for coffee planting framework in the category agroecological management system is recognized by the agroecological transition process and occurred when the farmer had adopted practices like green manuring, management of weeds or the implementation of agroforestry and reducing the use of agrochemicals, especially pesticides [3,5,6]; N/A = Not applicable.

The sites used in this study had permission by the owner of the land to collect soil samples. All historic use information were gave us by the owner and by literature [41]. The forest fragment also belonged to the same private owners.

### Sampling and soil characterization

Samples were taken over three different periods within each site that corresponded to the phenological cycle of coffee: 1^st^ (flowering–Nov 2012), 2^nd^ (grain filling–March 2013), and 3^rd^ (harvesting–July 2013). Three sampling points, distant from each other by at least 10 m, were selected within each site and period. In each sampling point, 5 soil subsamples were obtained (depth 0–20 cm) and pooled, totaling 3 samples per site in each period making a total of 81 samples for the study. Samples were divided into two portions. One was stored at 4°C for DNA and spore extraction and the other portion was sent for analysis of soil physical [42] and chemical properties [43–46].

### Mycorrhizal colonization

Roots were manually picked at the same points of soil sampling from the base of the coffee stem, certifying that these roots belonged to coffee plant and not of spontaneous plants, washed, and preserved in FAA (formaldehyde: alcohol: acetic acid; 90: 5:5, v:v:v). For staining, roots were immersed in 10% KOH (w:v) for 6–10 days at room temperature and washed in tap water to remove excess KOH. Roots were then immersed in H_2_O_2_ 30% (10 min), washed in tap water, and acidified in 1% HCl (v:v) for 5 min. After discarding the HCl, roots were stained with trypan blue 0.05% in lactoglycerol (w:v) for 12 h at room temperature (adapted from [47] Stained roots were stored in lactoglycerol (w:v) [48]. Mycorrhizal colonization was evaluated using the grid line intersection method [49] by observing 300 points of intersection between the root fragments and crossing lines in a Petri dish under a dissecting microscope.

### Extraction, quantification and morphospecies identification

From each sample, 100 cm^3^ of soil were used to extract AMF spores by wet sieving [50], followed by centrifugation in water and 50% sucrose solution. The total number of AMF spores were counted under a dissecting microscope.

Spores were separated by morphotypes based on spore color, size and type of spore formation (acaulosporoid, glomoid, or gigasporoid), and mounted on slides using polyvinyl-lacto-glycerol (PVLG) or with PVLG + Melzer (1:1, v:v). Morphological identification was carried out by observing spore wall characters (*e*.*g*. wall thickness, ornamentations, Melzer’s reaction) and making comparisons with descriptions of AMF reference cultures of the International Culture Collection of (Vesicular) Arbuscular Mycorrhizal Fungi (http://invam.caf.wvu.edu) and Blaszkowski [51]. Nomenclature for AMF genera and classification follows [52] Redecker *et al* (2013).

### Molecular analysis: PCR-DGGE and sequencing

From the spores obtained in 50 cm^3^ of soil, by the technique of wet sieving, total DNA was extracted with Power Soil DNA Kit, Mobio (Mobio Laboratories, Carlsbad, CA, USA), according to the manufacturer's guidelines and small adjustments in the following steps of the protocol: (1) use of 600 μl of the spore suspension; (7) transfer of 700 μl of the supernatant for the collection tube; (10) transfer of 750 μl of the supernatant to the collection tube.

The DNA extracted from the three samples from each area was mixed. After that, PCR reaction was done in triplicate. The GoTaq Flex DNA Polymerase (Promega, Madison, USA) in 50 μl buffer (20 mM Tris-HCl; 50 mM KCl; pH 8.4) was used following the manufacturer's recommendations. To 3 μl of DNA, four triphosphate desoxinucleosídeos (200 μM); MgCl_2_ (1.5 mM), two primer (0.2 μM); enzyme GoTaq Flex DNA polymerase (1.25 U) and acetylated bovine serum albumin (0.8 μg μl^-1^—BSA, Promega) were added to optimize the action of the polymerase. The first DNA amplification of the 18S rDNA fraction corresponded to arbuscular mycorrhizal fungi, with AM1 primer (5'GTTTCCCGTAAGGCGCCGAA-3') [53] combined with the primer NS31 (5'TTGGAGGGCAAGTCTGGTGCC -3 ') to obtain fragments of 560 bp [54].

The amplification of DNA fragments by PCR took place in a thermocycler (Mastercycle Epgradient Eppendorf) by taking the following steps: a) a first cycle of 1 min at 94°C, 1 min to 66°C and 1.5 min at 72°C; b) 30 cycles of 30s at 94°C, 1 min to 66°C and 30 s at 72°C and c) final extension of 10 min at 72°C. Confirmation of the amplified products was determined with 5 μl of the PCR reactions by submission to electrophoresis on agarose gel 0.8% (w:v), staining with ethidium bromide, and visualization under UV light in the photodocumentation imaging system (Loccus Biotecnologic L-Pix Chemi).

For the second amplification, nested PCR, we attempted to obtain smaller DNA fragments, about 230 bp, to perform the gel electrophoresis denaturing gradient (DGGE), using 1 μl of the product of the first PCR reaction, previously diluted in nine microliters of sterilized MilliQ water. The primers were NS31-GC (5’-CGCCCGGGGCGCGCCCCGGGCGGGGCGGGGGCACGGGGGTTGGAGGGCAAG TCTGGTGCC-3’) [55] and Glo1 (5’-GCCTGCTTTAAACACTCTA-3’) [56]. For the nested PCR reaction the same mixture as described for the first PCR reaction was used. Amplification took place in a thermocycler under the following stages: a) initial DNA denaturation for 5 min at 94°C; b) followed by 35 cycles with 45 s denaturation at 94°C; c) pairing by 45 s at 52°C and extension for 1 min at 72°C. The products were checked as previously described. This product was used for DGGE analysis (Model System DCodeTM—Bio-Rad California USA) according to the method described by Liang *et al*. [37], with minor modifications. Polyacrylamide gel (37.5: 1 acrylamide: bisacrylamide) to 8% (w:v) was used in a Tris-acetate-EDTA buffer (TAE) 1X (Tris/acetic/acid EDTA, pH 8.0). The gradient was obtained with the trainer gradient SG50 Hoefer (Amersham Biosciences) and by mixing two stock solutions of polyacrylamide, and 100% denaturing urea composed of 7 mol l^-1^ (Sigma, cat # U5378) and formamide 40% (v:v) (Sigma, Cat # F9037) and 0% denaturation without these reagents. The final gradient gel obtained by mixing the solutions ranged from 35 to 50%.

Electrophoresis was applied using 1X TAE buffer at a constant temperature of 60°C to 80 V for a period of 10 min, followed by 60 V for 20 h. Gels were stained after completion of electrophoresis for 30 min in 1X SYBR GOLD solution (Sigma Aldrich) according to the manufacturer’s recommendations. Images of the gels were observed by photodocumentation as previously described.

The images were analyzed using the Bionumerics software program, which allowed for the construction of dendrograma using the Sorensen-Dice index and cluster analysis by the method of minimum variance (Ward) to assess the similarity between the AMF communities, regarding the distance and the pattern of bands corresponding to the gene 18S rDNA AMF, according to the presence/absence of an amplified region.

The well-defined band with good intensity were selected, collected and transferred to 0.5 ml microtubes containing 30 μl of sterile miliQ water. These samples were subjected to new PCR amplification, similar to nested-PCR, without a GC clamp. The fragments were sequenced with primer Glo1 and NS31. The number of eluted bands ranged from five in the treatment A Z2 1^st^ and C Z3 2^nd^ to 28 in the treatment A Z1 2^nd^, totaling 356 eluted bands, which were sequenced. The sequencing allowed formation of 235 contigs with high quality, which were grouped into 114 operational taxonomic units (OTU), using 97% as the minimum identity by MOTHUR Software [57] and 103 has received taxonomy assigned to OTU using MaarjAM [58] as a database reference. Sequences of each OTU was deposited in GenBank under accession numbers MK305986—MK306099.

### Statistical analysis

The percentage of mycorrhizal colonization was determined after a Kolmogorov-Smirnov normality test. In order to assess the total abundance of spores the data were transformed using log. (X), followed by analysis in split plots, considering management, locality and period, and means were compared by Tukey test (p < 0.05). The data chemical characteristics of soil was submitted to ANOVA and means compared by Tukey test (p < 0.05). These analyses were carried out using the statistical program Assistat 7.7.

Similarity of morphotypes was compared among systems, localities and period by Sorensen Dice-index and cluster analysis using the method of minimum variance (Ward) for showing affinity of samples according to a morphospecies composition using the PAST software.

The pattern of similarity and intensity of bands present in the DGGE gels were evaluated by the Sorensen Dice-index, followed by cluster analysis using the method of minimum variance (Ward) for building dendrograms, Bionumerics 5.1. For principal component analysis (PCA), the similarity matrix was used along with the chemical characteristic of the soil, using the Euclidean distance and logarithmic transformation of the data (log. (X + 1) by Canoco version 4.5 (Biometris, Wageningen, The Netherlands).

## Results

### Mycorrhizal colonization

**Fig 1:**
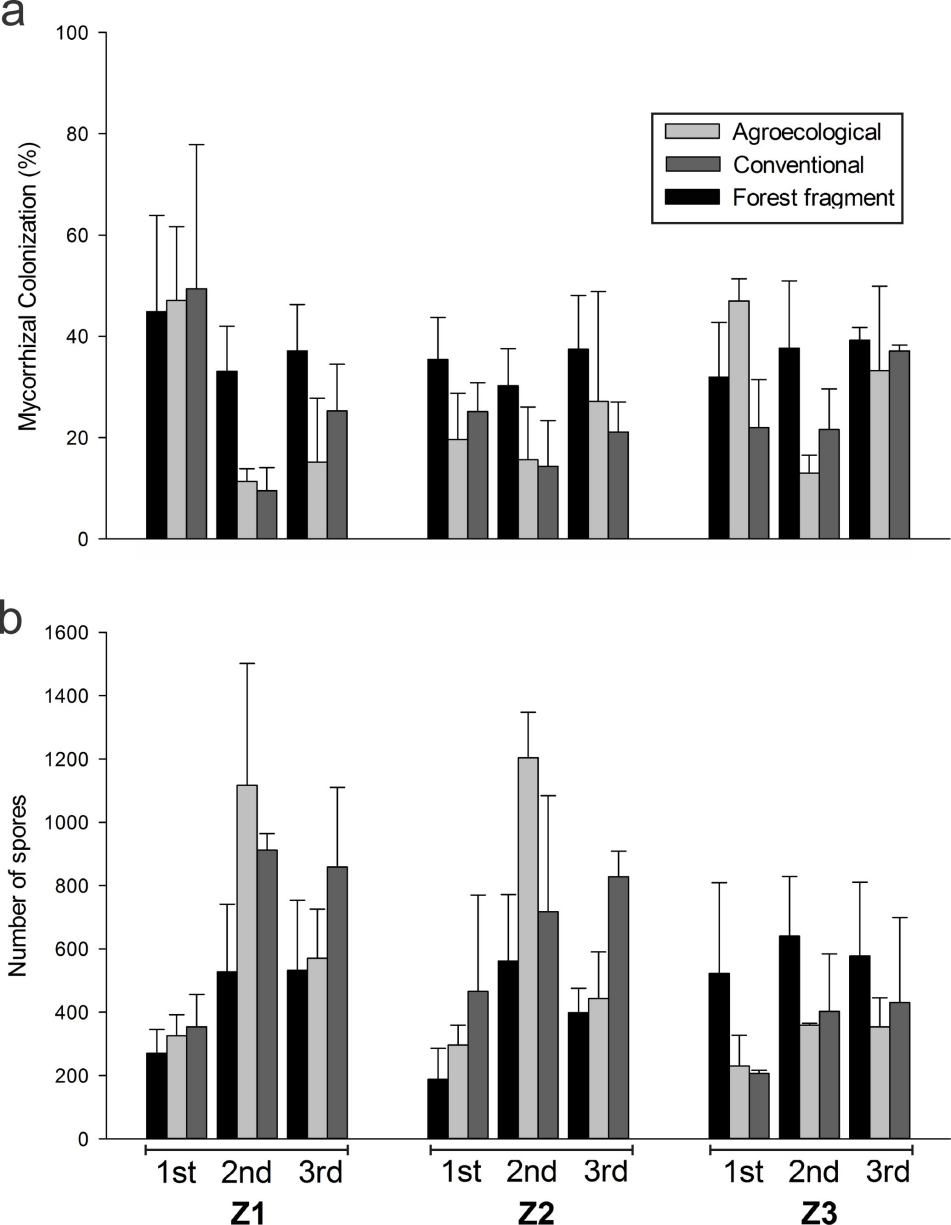
(a) Percentage of colonization of the forest fragment species and agroecological and conventional coffee root, in three localities (Z1, Z2 and Z3) and three periods, 1st (flowering), 2nd (grain filling) and 3rd (harvesting). Bar = standard deviation. (b) Number of spores of the forest fragment and agroecological and conventional coffee soil, in three localities (Z1, Z2 and Z3) and three periods, 1st (flowering), 2nd (grain filling) and 3rd (harvesting). Bar = standard deviation.

No differences were observed between the percentages of mycorrhizal colonization of the conventional and agroecological management systems, but in general, these management styles had lower colonization than forest fragment (Fig 1A, Tables A-C in S1 File). The percentage of colonization was higher in the flowering and harvesting period than in the grain filling period and site Z1 had the highest average percentage of colonization during the flowering period (Fig 1A, Tables A-C in S1 File).

### Number of spores

Overall, AMF spore numbers differed between periods and localities, but not between management systems (Fig 1B, Tables D-F in S1 File). The number of spores was significantly lower in the flowering period relative to graining and harvesting, and lower in Z3 compared to Z2 and Z1 (Table E in S1 File). The number of spores in the agroecological and conventional management systems was significantly higher in Z1 and Z2 compared to the Z3 locality, while forest fragment showed the opposite result (Table F in S1 File).

### Morphospecies identification

A total of 42 AMF morphospecies was identified based on spore morphology, distributed over nine genera and six families of the main monophyletic clades of Glomeromycota phylum. Morphospecies pertained to genera *Acaulospora* (17), *Glomus* (16), *Ambispora* (1), *Archaeospora* (1), *Dentiscutata* (2), *Gigaspora* (1), *Paraglomus* (1), *Rhizophagus* (1), and *Scutellospora* (3) were detected. No differences in richness of species between management systems, localities or periods were detected (Table G in S1 File), but forest fragment and Z1 had a higher number of exclusive morphospecies (Tables H and I and Fig A in S1 File).

**Fig 2:**
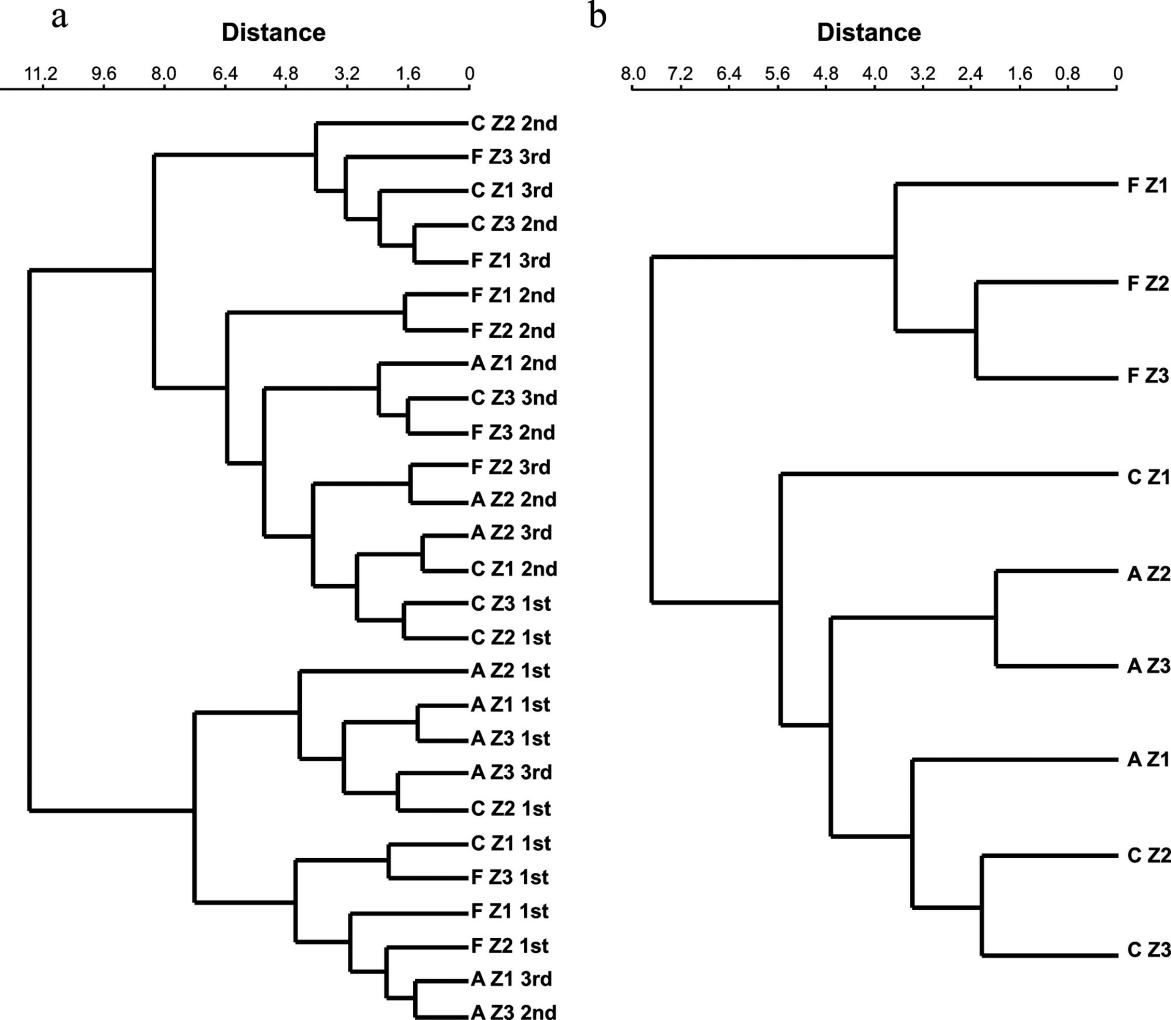
**Dendrogram of similarity of morphospecies of arbuscular mycorrhizal fungi of samples (a) per management, localities and period, and (b) per management and localities (b)**. F = forest fragment; A = agroecological; C = conventional; Z1, Z2 and Z3 = localities; 1^st^ = flowering; 2^nd^ = grain filling; 3^rd^ = harvesting.

The dendrogram for the composition of morphospecies (Fig 2A) suggested management and locality might form a group composed mainly of samples from the 2^nd^ (grain filling) and 3^rd^ (harvesting) period, and a second group consisting of samples primarily from the 1^st^ (flowering) period. When the sampling period is omitted, the forest fragment forms a group distinct from the agroecological and conventional coffee management systems (Fig 2B). The locality Z1 presents less similarity with Z2 and Z3, due to the greater number of exclusive species (Fig 2B, Fig A and Table I in S1 File).

### PCR-DGGE, multivariate and sequence analysis

**Fig 3:**
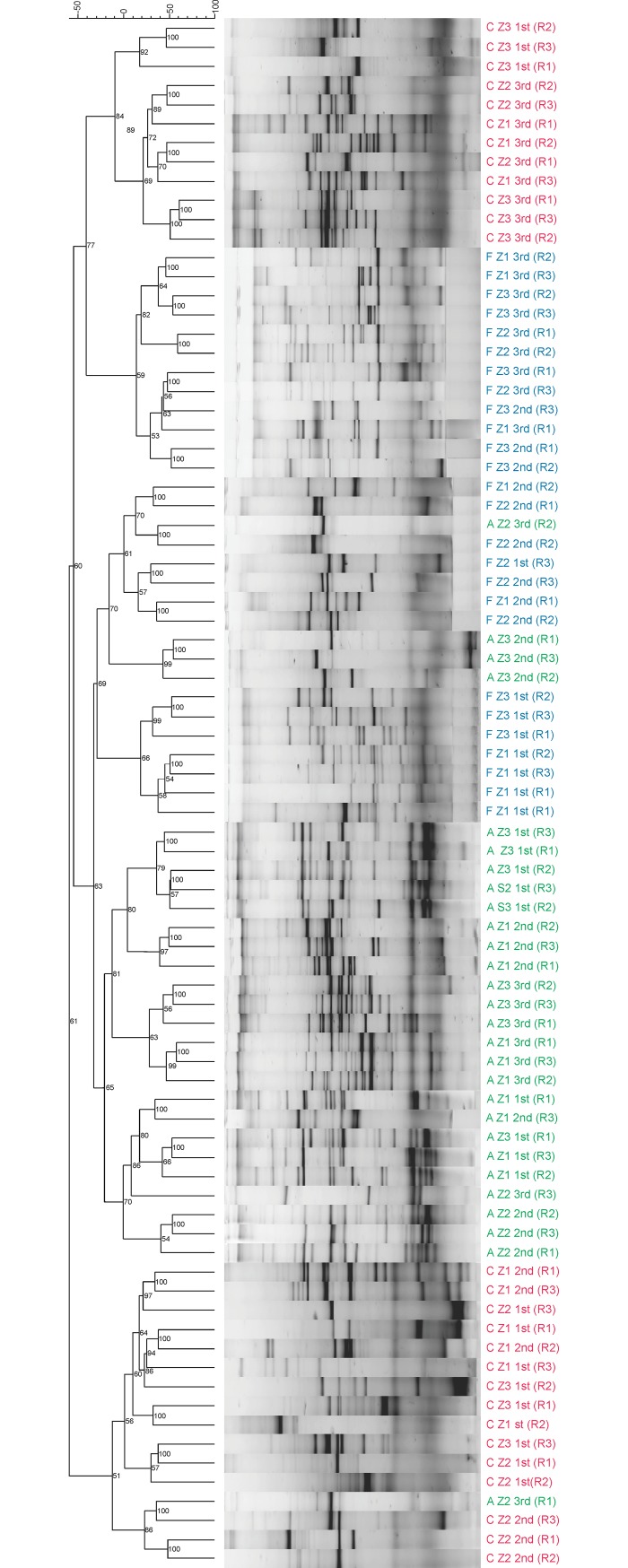
**Dendrogram obtained by the Sorensen-Dice calculated by the standard of bands obtained by PCR-DGGE demonstrating similarity in management, forest fragment (F) and agroecological (A) and conventional (C) coffee management, in three localities (Z1, Z2 and Z3) and three periods, 1st (flowering), 2nd (grain filling) and 3rd (harvesting). R = repetition**.

Analysis of the AMF community by PCR-DGGE technique allowed for assessing the community composition, using the Sorensen-Dice for dendrogram plotting (Fig 3). The cluster showed that management played a relevant role in the pattern of operational taxonomic unit (OTU) distribution. Formation of the groups varied according to locality and sampling period. The agroecological management of coffee system presented a higher degree of similarity to forest fragments, being that 96.3% of agroecological samples grouped with forest, while only 44.4% of conventional samples grouped with forest. In addition, 55.6% of samples from the conventional management did not cluster with the other samples (Fig 3). Most of Z2 conventional, that represents the most intense management (Table 1), were allocated to this group. Agroecological and conventional did not cluster, and the forest fragments were grouped with both.

The agroecological management of coffee system presented a higher diversity indices as compared to a conventional management system and does not differ from forest fragments (Table 2).The localities Z1 and Z2 presented OTU diversity indices higher than those of Z3 (Table 2). Four families (Acaulosporaceae, Diversisporaceae, Glomeraceae, Gigasporaceae) were identified by molecular methods, using the MaarjAM database and 11 OTU had no assigned identity (S2 File).

**Table 2:** Indices of diversity for OTU of arbuscular mycorrhizal fungi (AMF) obtained by the PCR-DGGE technique in forest fragment, and agroecological and conventional coffee, in three localities (Z1, Z2 and Z3) and three periods, 1st (flowering), 2nd (grain filling) and 3rd (harvesting).

	Richness	Chao	Dominance	Simpson	Shannon_H
**Management**					
**Forest fragment**	16.667 ab	155.222 ab	0.060 ab	0.921 ab	2.780 ab
**Agroecological**	19.148 a	207.259 a	0.064 b	0.940 a	2.898 a
**Conventional**	14.519 b	124.259 b	0.079 a	0.936 b	2.613 b
**Localities**					
**Z1**	18.074 a	183.815 a	0.061 b	0.940 a	2.852 a
**Z2**	14.333 b	123.630 b	0.082 a	0.920 b	2.588 b
**Z3**	17.926 a	179.296 a	0.060 b	0.940 a	2.851 a
**Period**					
**1**^**st**^	15.889 a	148.44 a	0.073 a	0.926 a	2.696 a
**2**^**nd**^	15.889 a	146.074 a	0.069 a	0.930 a	2.717 a
**3**^**rd**^	18.556 a	192.222 a	0.059 a	0.940 a	2.878 a

The averages followed by the same lowercase (columns) letter do not differ by Tukey test at 5% probability.

**Fig 4:**
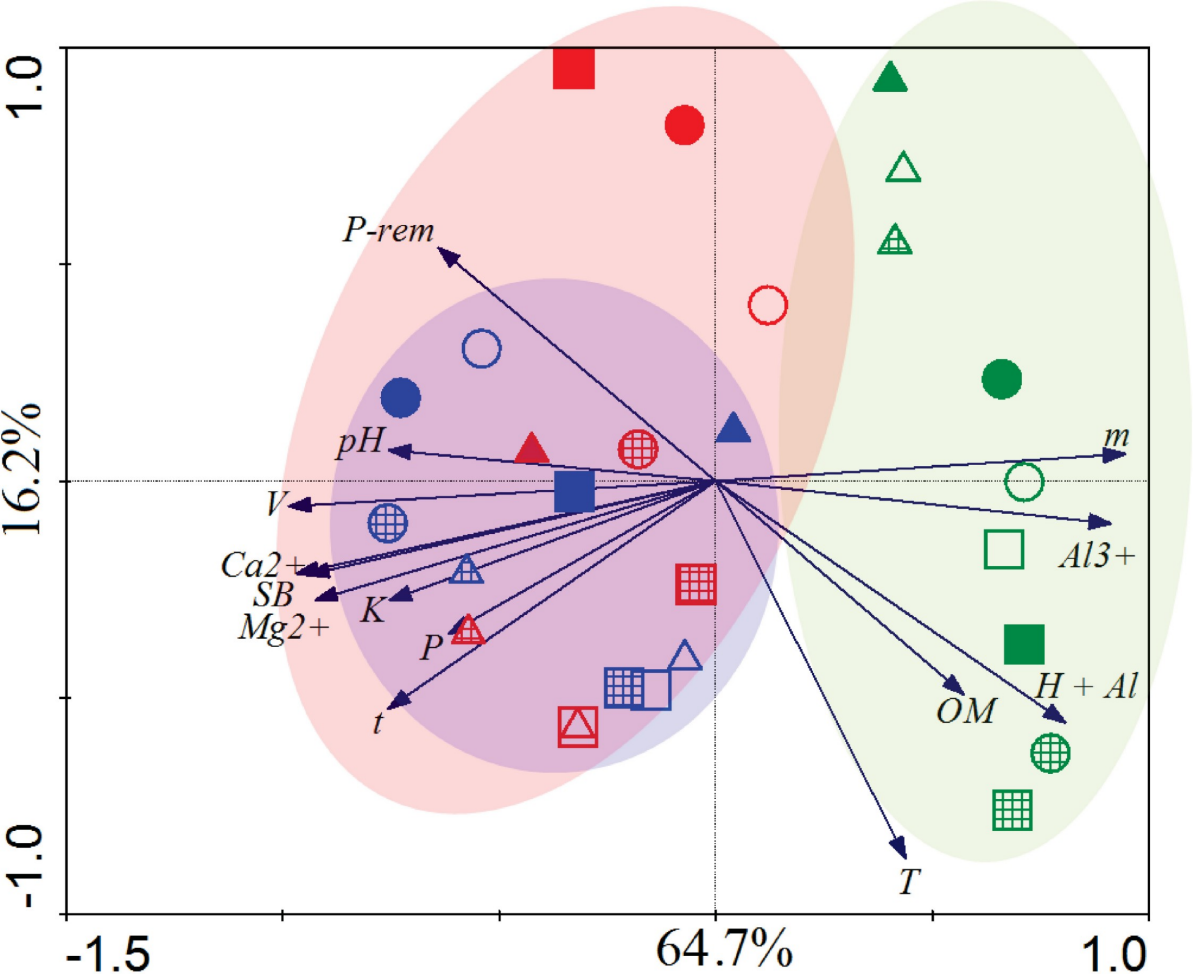
Principal Component Analysis (PCA) based on PCR-DGGE profiles for the AMF community and soil chemical attributes in management, forest fragment (green) and agroecological (blue) and conventional (red) coffee management systems, in three localities, Z1 (triangle), Z2 (circle) and Z3 (square) and three periods, 1st (flowering—solid), 2nd (grain filling–wide cross-line) and 3rd (harvesting–empty). SB = Sum of exchangeable bases; t = effective cation exchange capacity; T = cation exchange capacity at pH 7,0; V = base saturation index; m = aluminum saturation index; OM = organic matter.

Analyzing PCA (Fig 4), the difference in the composition of AMF community between plots was explained by ordination: the first (PC1) and the second (PC2) may account for 64.7 and 16.2% in variation, respectively. Soil chemical characteristics indicate a number of differences between localities and between management systems, in particular between coffee plantation and forest fragments (Table 3). So, the PCA analysis (Fig 4) revealed that forest fragments distinguished from coffee cultivation, especially in relation to organic matter (OM), levels of Al^3+^ and H + Al, and aluminum saturation index (m). The sum of exchangeable bases (SB), the remaining phosphorus (P-rem), P, K, bases saturation index (V), Ca^2+^, Mg^2+^, effective cation exchange capacity (t) contributed to distinguishing the AMF community between coffee management from forest fragments (Table 3).

**Table 3:** Statistical analysis of characteristics chemicals of soil (0–20 cm deep) in forest fragment (F), and agroecological (A) and conventional (C) management, in three localities (Z1, Z2 and Z3). Media from three period of annual coffee cycle.

Management	Locality	pH	P	K	Ca^+2^	Mg^+2^	Al^+3^	H + Al	SB	t	T	V	m	OM	P-rem
**F**	**Z1**	4.28 b	1.56 b	36.33 d	0.19 d	0.14 cd	1.52 ab	9.17 c	0.43 c	1.95 b	9.60 c	4.36 de	78.45 a	4.14 c	13.43 ab
	**Z2**	4.29 b	2.08 b	29.11 d	0.12 d	0.08 d	1.87 a	13.75 ab	0.27 c	2.15 ab	14.02 ab	2.02 e	86.59 a	6.94 ab	8.08 b
	**Z3**	4.44 ab	2.60 b	33.78 d	0.17 d	0.14 cd	1.72 a	15.07 a	0.39 c	2.11 ab	15.54 a	2.58 e	81.59 a	7.99 a	6.71 b
**A**	**Z1**	4.99 ab	7.30 ab	149.2 ab	1.26 c	0.42 abcd	0.79 bcd	9.80 bc	2.70 a	2.84 ab	11.87 abc	17.62 bc	30.26 bc	5.01 bc	13.2 ab
	**Z2**	5.12 a	25.80 a	62.56 cd	2.43 a	0.68 a	0.30 d	6.83 c	3.27 a	3.56 a	10.10 bc	32.14 a	9.70 c	3.83 c	24.34 a
	**Z3**	4.96 ab	3.96 ab	166.6 a	1.53 bc	0.41 abcd	0.59 cd	9.75 bc	2.37 ab	2.96 ab	13.02 abc	19.30 bc	20.49 c	4.93 bc	10.20 ab
**C**	**Z1**	5.24 a	5.67 ab	118.6 abc	2.16 ab	0.61 ab	0.55 cd	8.90 c	3.07 a	3.62 a	11.96 abc	26.43 ab	15.48 c	5.32 bc	15.07 ab
	**Z2**	4.77 ab	3.51 b	84.4 bcd	0.89 cd	0.31 bcd	1.29 abc	8.34 c	1.42 bc	2.70 ab	9.76 bc	14.14 cd	49.61 b	3.32 c	13.74 ab
	**Z3**	5.19 a	3.50 b	97.3 abcd	1.40 bc	0.48 abc	0.63 bcd	9.12 c	2.12 ab	2.76 ab	11.24 abc	19.59 bc	22.58 c	4.54 c	11.30 ab

Extractors used: P, K, Zn, Cu = Extrator Mehlich1; Al^3+^, Ca^2+^ e Mg^2+^ = Extractor KCl 1 mol.L^-1^; H + Al = Extractor acetate de Ca 0.5 mol.L^-1^ SB = Sum of exchangeable bases; t = Effective cation exchange capacity; T = Cation exchange capacity at pH 7,0; V = base saturation index; m = aluminum saturation index; OM = organic matter; The data followed by the same lowercase letter, in the same collum, do not differ by Tukey test at 5% probability

## Discussion

In this study, we assessed the AMF community based on morphological and molecular methods under two coffee management systems and compared them with natural forest. We sampled three different periods corresponding to distinct phenological states of coffee plants in three areas. Overall, root colonization but not spore numbers was affected by the system of management. Data obtained with ecological indices corroborated our hypothesis that AMF communities under agroecological coffee plantations has more richness and diversity than conventional plantations (Table 2). This result suggests that an agroecological coffee system is important to maintaining a diverse AMF community and representing a management system that should be adopted.

Mycorrhizal colonization was affected mainly by management systems and periods. Lower levels of coffee root colonization in both agroecological and conventional management were detected compared to forest area (Fig 1A). It is suggested that this lower percentage of colonization in coffee plants is related to the higher P content in the soil, as P-rem values under coffee systems was higher than in the forest (Table 3). Phosphorus is a nutrient involved in the nutritional ecology of AMF and may influence mycorrhizal colonization [23,59]. Furthermore, forest fragments present a higher diversity and abundance of plant species in different phenological stages compared to coffee plantations, which provide different niche conditions for colonization over time [21,60]. The percentage of mycorrhizal colonization was affected by the period (Fig 1B Tables A-C in S1 File) indicating that the change in the AMF external mycelium network may be a response to the season [60,61] since a lower carbon amount is allocated to symbionts during the coffee grain filling, which may modulate AMF diversity [62,63]. The difference in root colonization between localities Z1 and Z2 during flowering may be related to local factors, which can select species with distinct symbiotic habits that influence the degree of root colonization.

Conversely, the number of AMF spores was influenced by phenological states and locality but not by management systems (Fig 1B and Tables D-F in S1 File). The sporulation increased during grain filling (2^nd^ period–Fig 1B and Table F in S1 File), corroborating the results related to temporal variations in the number of spores in coffee plantation [21,64] and forest [21,60,64]. During the flowering of coffee (rainy season), AMF presents a vegetative growth, while the number of spores decreases due to germination [65], whereas in the grain filling and harvesting (dry season), environmental factors such as low soil moisture or the phenological stage of plants promote spore formation [21,60].

We detected a diverse AMF community associated with coffee plantation and forest system, comprising nine genera and six families. The richness of species by morphology did not differ from any one management system, locality nor period to another (Table H in S1 File). AMF species richness is similar to that found for coffee cultivation in Mexico [21], Colombia [25], and Ethiopia [26,32]. In all these studies and herein, *Acaulospora* and *Glomus* were the most representative genera in the AMF community. This result contrasts with that of Fernandes *et al*. [27] who detected a higher number of Gigasporaceae in coffee plantation. Nevertheless, for the management, localities or periods, no difference was observed in the richness of morphospecies (Table H in S1 File). The composition of morphospecies is different due to the occurrence of particular species in different localities and period of sampling (Fig A and Table I in S1 File). Therefore, sampling in two or more periods, according to the season and plant cycle (Fig 2A) is necessary, as shown by Hart *et al*. [66].

The number of AMF species varies, whether it is evaluated by morphological or molecular biological methods [52,67]. Therefore, it is necessary to associate classical taxonomic evaluations with molecular biological techniques, such as PCR-DGGE [68] and/or sequencing [22,36,69], to more thoroughly assess AMF community diversity. The families Ambisporaceae, Archeosporaceae and Paraglomeraceae were not detected by sequencing, while Diversisporaceae was not detected by morphology. (Table G in S1 File and S2 File).

Our results with DGGE indicate that coffee management may impact AMF community on a local scale (Fig 3), as conventional coffee plantations apart from forest and agroecological coffee tended to form two distinct clusters. Similar results have been observed in horticulture farms [38] where conventional management has a negative impact on mycorrhizal fungi compared to organic management. The higher number of OTU exclusive (Fig 4) and diversity indices (Table 2) in agroecological management may be related to the availability of niches [70] due to the heterogeneity which increases the complexity of the system, since the resources, such as light and host plant are similar to forest fragment, and soil chemical characteristics such as pH, P and OM are similar to conventional management system because of the fertilization (Table 3). Agroecological management practices can minimize the negative impacts of agriculture, by decreasing competition between AMF species [71] and the toxic effects of agrochemicals [11]. In addition, monoculture and fertilization can select less efficient species and decrease AMF diversity [20,72,73], mainly in annual crops [17,34]. Management might interfere in community composition, without necessarily reducing spore number, mycorrhizal colonization or AMF species richness.

Maintenance of forest fragments, free from inputs and other agricultural practices is as important as agroecological management, to maintain specific AMF groups. Furthermore, this study indicated that localities influence the diversity of AMF (Table 2). Further studies need to consider the biogeography evaluation of AMF [74], as well as to know the influence of microclimatic, edaphic and phytosociological factors in space and time on the community composition of AMF.

## Conclusions

This study contributes to the understanding the impacts of the system of agricultural management on AMF and provides evidence that agroecology represents a promising management approach for the implementation of sustainable agriculture. Molecular analysis showed that agroecological system maintains wider AMF diversity compared to conventional systems, although there is no difference in the abundance of spores, roots colonization nor richness of AMF by morphology. The cycle of coffee and locality affect the composition of AMF community, showing the importance of considering plant phenology, and spatial scale for sampling.

## Supporting information

S1 File Table A. ANOVA of percentage of mycorrhizal colonization of roots of forest fragment species and agroecological and conventional coffee roots in three periods and three localities using split plot experimental design. Table B. Percentage of colonization of the root of forest fragment species and agroecological and conventional coffee roots in three localities(Z1, Z2 and Z3) and three periods, 1st (flowering), 2nd (grain filling) and 3rd (harvesting). Table C. Percentage of colonization of the root of forest fragment species and agroecological and conventional coffee roots for interaction between periods and localities. Table D. ANOVA of number of spores of the forest fragment and agroecological and conventional coffee soil in three periods and three localitiesusing split plot experimental design. Table E. Number of spores in the forest fragment and agroecological and conventional coffee soil in three periods and three locations. Table F. Number of spores in the forest fragment and agroecological and conventional coffee soil in three localities for interaction among management and locations. Table G. Morphospecies of arbuscular mycorrhizal fungi (AMF) found in forest fragment, and agroecological and conventional coffee soil, in three localities (Z1, Z2 and Z3) and three periods, 1st (flowering), 2nd (grain filling) and 3rd (harvesting). Table H. Richness of morphospecies of arbuscular mycorrhizal fungi (AMF) found in forest fragment and agroecological and conventional coffee soil, in three localities (Z1, Z2 and Z3) and three periods, 1st (flowering), 2nd (grain filling) and 3rd (harvesting). Table I. Morphospecies of arbuscular mycorrhizal fungi (AMF) found exclusively or shared among forest fragment (F) and agroecological (A), and conventional (C) coffee in three localities (Z1, Z2 and Z3) and three periods, 1st (flowering), 2nd (grain filling) and 3rd (harvesting). Fig A. Venn diagram of arbuscular mycorrhizal fungi, showing number of morphospecies exclusively or shared among management [(a): F = forest fragment; A = agroecological; C = conventional], localities [(b): Z1; Z2; Z3] and period [(c): 1st = flowering; 2nd = grain filling; 3rd = harvesting], generated using an online tool (http://bioinformatics.psb.ugent.be/webtools/Venn/).
(PDF)

S2 File Assigned taxonomy to OTU of arbuscular mycorrhizal fungi (AMF) obtained by sequencing of PCR-DGGE bands, found among forest fragment (F), agroecological (A), and conventional (C) coffee in three localities (Z1, Z2 and Z3) and three periods, 1st (flowering), 2nd (grain filling) and 3rd (harvesting). (XLSM)

S3 File Summary statistics: Sheet 1) Mycorrzal colonization; Sheet 2); Number of spores; Sheet 3) Morphospecies identification; Sheet 4) Bands intensity–DGGE; Sheet 5) Index–DGGE; Sheet 6) Chemicals of soil. Forest fragment (F), agroecological (A), and conventional (C) coffee in three localities (Z1, Z2 and Z3) and three periods, 1st (flowering), 2nd (grain filling) and 3rd (harvesting). R **=** repetition.
(XLSX)
