# Supplementary material for: Agroecological coffee management increases arbuscular mycorrhizal fungi diversity
Source: PLoS One. 2019 Jan 8;14(1):e0209093. doi: 10.1371/journal.pone.0209093 (PMC6324804; doi:10.1371/journal.pone.0209093)
Supplement: S1 File — Table A. ANOVA of percentage of mycorrhizal colonization of roots of forest fragment species and agroecological and conventional coffee roots in three periods and three localities using split plot experimental design. Table B. Percentage of colonization of the root of forest fragment species and agroecological and conventional coffee roots in three localities(Z1, Z2 and Z3) and three periods, 1st (flowering), 2nd (grain filling) and 3rd (harvesting). Table C. Percentage of colonization of the root of forest fragment species and agroecological and conventional coffee roots for interaction between periods and localities. Table D. ANOVA of number of spores of the forest fragment and agroecological and conventional coffee soil in three periods and three localitiesusing split plot experimental design. Table E. Number of spores in the forest fragment and agroecological and conventional coffee soil in three periods and three locations. Table F. Number of spores in the forest fragment and agroecological and conventional coffee soil in three localities for interaction among management and locations. Table G. Morphospecies of arbuscular mycorrhizal fungi (AMF) found in forest fragment, and agroecological and conventional coffee soil, in three localities (Z1, Z2 and Z3) and three periods, 1st (flowering), 2nd (grain filling) and 3rd (harvesting). Table H. Richness of morphospecies of arbuscular mycorrhizal fungi (AMF) found in forest fragment and agroecological and conventional coffee soil, in three localities (Z1, Z2 and Z3) and three periods, 1st (flowering), 2nd (grain filling) and 3rd (harvesting). Table I. Morphospecies of arbuscular mycorrhizal fungi (AMF) found exclusively or shared among forest fragment (F) and agroecological (A), and conventional (C) coffee in three localities (Z1, Z2 and Z3) and three periods, 1st (flowering), 2nd (grain filling) and 3rd (harvesting). Fig A. Venn diagram of arbuscular mycorrhizal fungi, showing number of morphospecies exclusively or [file pone.0209093.s001.pdf]

Table A.

Source of Variation	Degrees of freedom	Sum of Square	Medium Square	F
Management	2	2211.40	1105.70	11.81 **
Residue	6	561.69	93.62	
Plots	8	2773.09		
Period	2	3157.09	1578.54	10.87 **
Management X Period	4	1067.94	266.98	1.84 ns
Residue	12	1742.12	145.18	
Subplots	26	8740.24		
Locality	2	610.20	305.10	2.21 ns
Management X Locality	4	273.85	68.46	0.49 ns
Periods X Locality	4	2056.59	514.14	3.72 *
Management X Periods X Locality	8	1448.54	181,068	1.31 ns
Residue	36	4966.06	137,95	
**Total**	80	18095.48		

** Significant at 1% probability (p <0.01) by Tukey test; * Significant at 5% probability (= 0.01 <P <0.05) by Tukey test ns not significant (p> = 0.05)

Table B.

Management	Colonization %	Period	Colonization %	Locality	Colonization %
**Forest fragment**	36.33 a	**Floration (1st)**	35.82 a	**Z1**	30.31 a
**Agroecological**	25.47 b	**Graining (2nd)**	20.71 b	**Z2**	25.11 a
**Conventional**	25.04 b	**Harvest (3rd)**	30.30 a	**Z3**	31.41 a

The averages followed by the same lowercase letter do not differ by Tukey test at 5% probability.

Table C.

Period	Locality		
	**Z1**	**Z2**	**Z3**
	Colonization %	Colonization %	Colonization %
**Floration (1st)**	47.12 aA	26.72 aB	33.62 abA
**Graining (2nd)**	17.99 bA	20.07 aA	24.08 aA
**Harvest (3rd)**	25.82 bA	28.56 aA	26.52 aA

*See details in supplementary 1. Mean followed by the same lowercase letter (columns) and upper (rows) do not differ by Tukey test at 1 % probality.

Table D.

Variation source	Degree of freedom	Sum of Squares	Medium square	F
Systems	2	0.07	0.0346	1.28 ns
Residue	6	0.16	0.02705	
Plots	8	0.23		
Period	2	1.75	0.87	38.29 **
Residue	12	0.27	0.02	
SubPlots	26	2.43		
Locality	2	0.35	0.17	6.74 **
Management X Locality	4	0.87	0.22	8.38 **
Periods X Locality	4	0.16	0.04	1.57 ns
Management X Periods X Locality	8	0.19	0.02	0.96 ns
Residue	36	0.92	0.03	
**Total**	80	4.93		

**Significant at 1% probability (p <0.01) by Tukey test

*Significant at 5% probability (= 0.01 <P <0.05) by Tukey test ns not significant (p> = 0.05))

Table E.

Management	Nº of spores	Period	Nº of spores	Locality	Nº of spores
**Forest fragment**	468.59 a	**Floration (1st)**	317.48 b	**Z1**	607.41 a
**Agroecological**	544.33a	**Graining (2nd)**	715.70 a	**Z2**	566.93 a
**Conventional**	575.04 a	**Harvest (3rd)**	554.78 a	**Z3**	413.63 b

The averages followed by the same lowercase letter do not differ by Tukey test at 5% probability. To meet the assumption of normality data were transformed to log. (X).

Table F.

Management	Locality*		
	Z1	Z2	Z1
	Nº of spores	Nº of spores	Nº of spores
**Forest fragment**	407.38 bAB	331.13bB	549.54 aA
**Agroecological**	575.44 abA	537.03 aA	302.00 bB
**Conventional**	645.65 aA	602.56 bB	309.03 bB

*See details in Supplementary 01. Mean followed by the same lowercase letter (columns) and upper (rows) do not differ by Tukey test at 5% probability

Table G.

Morphospecies	Forest fragment									Agroecological									Conventional								
	Z1			Z2			Z3			Z1			Z2			Z3			Z1			Z2			Z3		
	1st	2nd	3rd	1st	2nd	3rd	1st	2nd	3rd	1st	2nd	3rd	1st	2nd	3rd	1st	2nd	3rd	1st	2nd	3rd	1st	2nd	3rd	1st	2nd	3rd
Acaulosporaceae																											
Acaulospora denticulata Sieverd. & S. Toro.																									X		
Acaulospora foveata Trappe & Janos					X				X					X													
Acaulospora herrerae Furrazola, B.T. Goto, G.A. Silva, Sieverd. & Oehl								X			X	X									X		X				
Acaulospora mellea Spain & N.C. Schenck.	X	X		X	X	X	X	X	X	X	X	X	X	X	X	X	X	X	X	X	X	X	X	X	X	X	X
Acaulospora morrowiae Spain & N.C. Schenck.				X	X	X		X	X	X	X					X	X	X	X		X		X	X	X	X	
Acaulospora nivalis Oehl, Palenz., I.C. Sánchez, G.A. Silva & Sieverd.										X																	
Acaulospora scrobiculata Trappe											X	X	X			X	X	X	X	X	X		X		X		X
Acaulospora sieverding Oehl, Sýkorová & Błaszk.					X				X										X		X						
Acaulospora sp.1	X		X		X		X					X			X		X				X				X		X
Acaulospora sp.2	X	X		X	X	X	X	X	X																		
Acaulospora sp.3			X																								
Acaulospora sp.4								X				X	X													X	
Acaulospora sp.5																	X										
Acaulospora sp.6				X		X																		X			
Acaulospora tuberculata Janos & Trappe		X						X		X	X											X					
Acaulospora verna Błaszk.											X										X						
**Ambisporaceae**																											
Ambispora leptoticha (N.C. Schenck & G.S. Sm.) C.										X	X													X		X	

Walker, Vestberg & A. Schüßler																											
**Archeosporaceae**																											
Archaeospora trappei (R.N. Ames & Linderman) J.B. Morton & D. Redecker	X		X		X	X						X	X					X	X		X	X		X			X
**Gigasporaceae**																											
Dentiscutata biornata (Spain, Sieverd. & S. Toro) Sieverd., F.A. Souza & Oehl	X	X																	X								
Dentiscutata cerradensis Spain & J. Miranda ex Sieverd., F.A. Souza & Oehl																				X							
Gigaspora sp. 1					X					X							X										
Scutellospora pernambucana Oehl, D.K. Silva, N. Freitas & L.C. Maia	X				X		X																				
Scutellospora sp. 1					X															X	X						
Scutellospora sp. 2					X	X	X	X	X					X						X							
**Glomeraceae**																											
Glomus aggregatum N.C. Schenck & G.S. Sm.				X	X	X																		X		X	
Glomus fuegianum (Speg.) Trappe & Gerd.													X			X	X	X	X		X				X	X	
Glomus glomerulatum Sieverd.	X	X		X	X	X	X	X	X	X	X	X	X	X	X	X		X	X		X	X	X	X	X	X	X
Glomus sp. 1	X			X	X	X		X	X	X	X	X	X	X		X				X			X	X			
Glomus sp. 2	X																										
Glomus sp. 3	X						X	X																			
Glomus sp. 4	X						X										X										
Glomus sp. 5		X	X	X	X	X	X	X	X	X	X	X	X	X	X	X	X	X	X	X	X		X	X	X	X	X
Glomus sp. 6		X	X		X		X	X		X	X	X	X	X		X		X	X	X					X		
Glomus sp. 7			X	X	X	X	X	X	X			X	X	X		X	X	X		X	X		X		X		X
Glomus sp. 8			X			X	X																				
Glomus sp. 9			X																								
Glomus sp. 10																					X			X	X		
Glomus sp. 11				X				X	X				X			X	X						X				X
Glomus sp. 12																										X	

Glomus spinuliferum Sieverd. & Oehl													X														
Rhizophagus fasciculatus (Thaxt.) C. Walker & A. Schüßler 2010					X														X								
**Paraglomeraceae**																											
Paraglomus occultum (C. Walker) J.B. Morton & D. Redecker										X											X						
**TOTAL 42**	11	07	08	10	18	12	12	14	11	11	11	11	12	08	04	10	11	09	11	09	15	04	09	10	11	09	08

Table H.

Management		Period		Locality	
	Richness		Richness		Richness
**Forest fragment**	11.11a	**Floration (1st)**	10.66 a	**Z1**	10.55 a
**Agroecological**	9.66 a	**Graining (2nd)**	10.22 a	**Z2**	10.44 a
**Conventional**	9.55 a	**Harvest (3rd)**	9.77 a	**Z3**	9.66 a

The averages followed by the same lowercase (columns) letter in columns do not differ by Tukey test at 5% probability.

Table I.

Management	Total	Morphospecies
F, A, C	13	*Glomus* sp. 7. *Acaulospora mellea*. *Glomus glomerulatum*. *Glomus* sp. 11. *Archaeospora trappei*. *Acaulospora tuberculata*. *Glomus* sp. 1. *Glomus* sp. 5. *Acaulospora* sp.1. *Acaulospora morrowiae*. *Glomus* sp. 6. *Acaulospora* sp.4. *Acaulospora herrerae*
F, A	4	*Scutellospora* sp. 2. *Glomus* sp. 4. *Gigaspora* sp. 1. *Acaulospora foveata*
F, C	6	*Dentiscutata biornata*. *Acaulospora sieverding*. *Glomus agregatum*. *Rhizophagus fasciculatus*. *Scutellospora* sp. 1. *Acaulospora* sp.6
A, C	5	*Glomus fuegianum*. *Acaulospora scrobiculata*. *Paraglomus occultum*. *Ambispora leptoticha*. *Acaulospora verna*
F	8	*Acaulospora* sp. 2. *Scutellospora pernambucana*. *Acaulospora* sp.3. *Glomus* sp. 8. *Acaulospora denticulata*. *Glomus* sp. 3. *Glomus* sp. 9. *Glomus* sp. 2
A	3	*Glomus spinuliferum*. *Acaulospora nivalis*. *Acaulospora* sp.5
C	3	*Glomus* sp. 12. *Glomus* sp. 10. *Dentiscutata cerradensis*

Period	Total	Morphospecies
1st, 2nd, 3rd	14	*Glomus fuegianum*. *Glomus* sp. 7. *Acaulospora mellea*. *Glomus glomerulatum*. *Glomus* sp. 11. *Archaeospora trappei*. *Glomus agregatum*. *Scutellospora* sp. 2. *Ambispora leptoticha*. *Glomus* sp. 1. *Glomus* sp. 5. *Acaulospora morrowiae*. *Glomus* sp. 6. *Acaulospora* sp.4
1st, 2nd	7	*Scutellospora pernambucana*. *Dentiscutata biornata*. *Glomus* sp. 3. *Acaulospora tuberculata*. *Rhizophagus fasciculatus*. *Glomus* sp. 4. *Gigaspora* sp. 1
1st, 3rd	8	*Acaulospora* sp.2. *Glomus* sp. 8. *Glomus* sp. 10. *Acaulospora sieverding*. *Acaulospora scrobiculata*. *Paraglomus occultum*. *Acaulospora* sp.1. *Acaulospora* sp.6
2nd, 3rd	4	*Scutellospora* sp. 1. *Acaulospora verna*. *Acaulospora foveata*. *Acaulospora herrerae*
1st	4	*Glomus spinuliferum*. *Acaulospora denticulata*. *Acaulospora nivalis*. *Glomus* sp. 2
2nd	3	*Glomus* sp. 12. *Dentiscutata cerradensis*. *Acaulospora* sp.5
3rd	2	*Acaulospora* sp.3. *Glomus* sp. 9

Locality	Total	Morphospecies
Z1, Z2, Z3	22	*Glomus fuegianum*. *Acaulospora* sp.2. *Glomus* sp. 7. *Acaulospora mellea*. *Scutellospora pernambucana*. *Glomus glomerulatum*. *Glomus* sp. 8. *Glomus* sp. 10. *Archaeospora trappei*. *Acaulospora sieverding*. *Acaulospora tuberculata*. *Acaulospora scrobiculata*. *Scutellospora* sp. 2. *Ambispora leptoticha*. *Glomus* sp. 1. *Gigaspora* sp. 1. *Glomus* sp. 5. *Acaulospora* sp.1.

		Acaulospora morrowiae. *Glomus* sp. 6. *Acaulospora* sp.4. *Acaulospora herrerae*
Z1, Z2	2	*Rhizophagus fasciculatus*. *Scutellospora* sp. 1
Z1, Z3	2	*Glomus* sp. 3. *Glomus* sp. 4
Z2, Z3	3	*Glomus* sp. 11. *Glomus agregatum*. *Acaulospora foveata*
Z1	8	*Acaulospora* sp.3. *Dentiscutata biornata*. *Acaulospora nivalis*. *Dentiscutata cerradensis*. *Paraglomus occultum*. *Acaulospora verna*. *Glomus* sp. 9. *Glomus* sp. 2
Z2	2	*Glomus spinuliferum*. *Acaulospora* sp.6
Z3	3	*Glomus* sp. 12. *Acaulospora denticulata*. *Acaulospora* sp.5

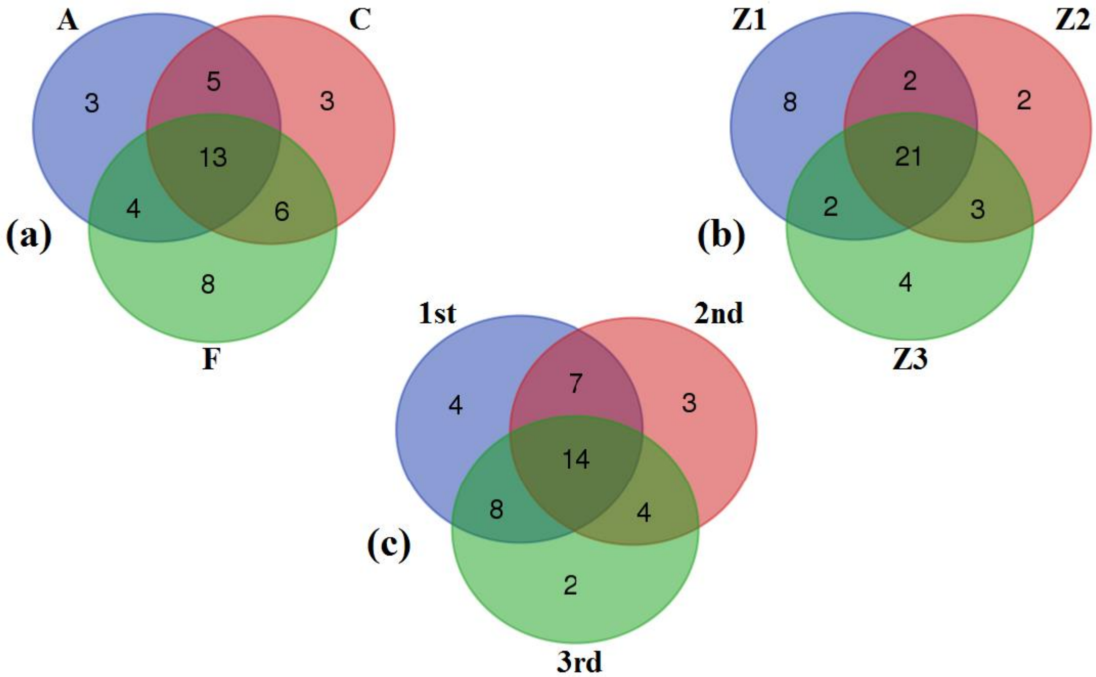

Fig A.
